# Supplementary figures and images for: Membrane contact sites regulate vacuolar fission via sphingolipid metabolism
Source: eLife. 2024 Mar 27;12:RP89938. doi: 10.7554/eLife.89938 (PMC10972560; doi:10.7554/eLife.89938)

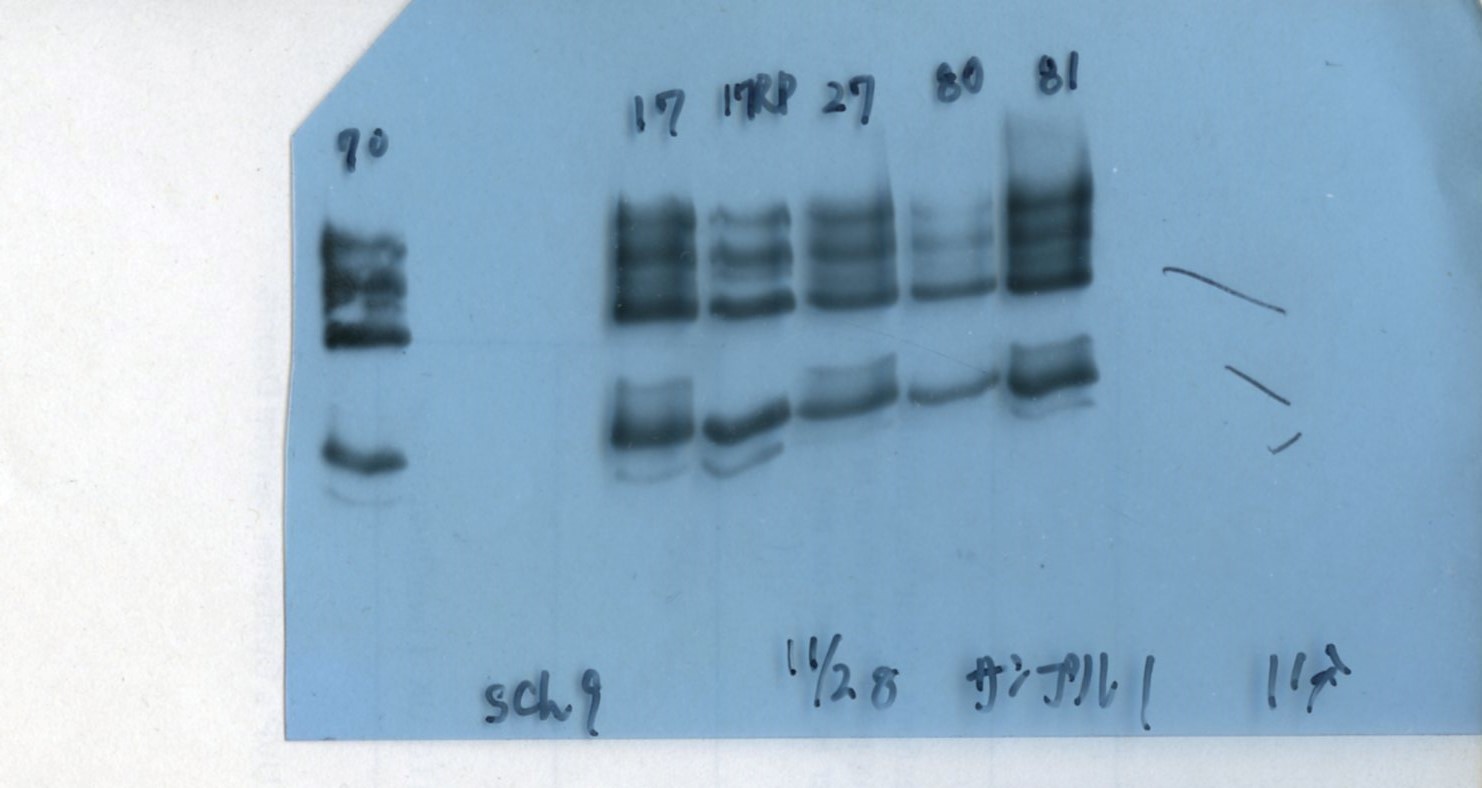

Supplement: Figure 1—source data 5. [file elife-89938-fig1-data5.zip › Figure 1-source data 5.jpg]

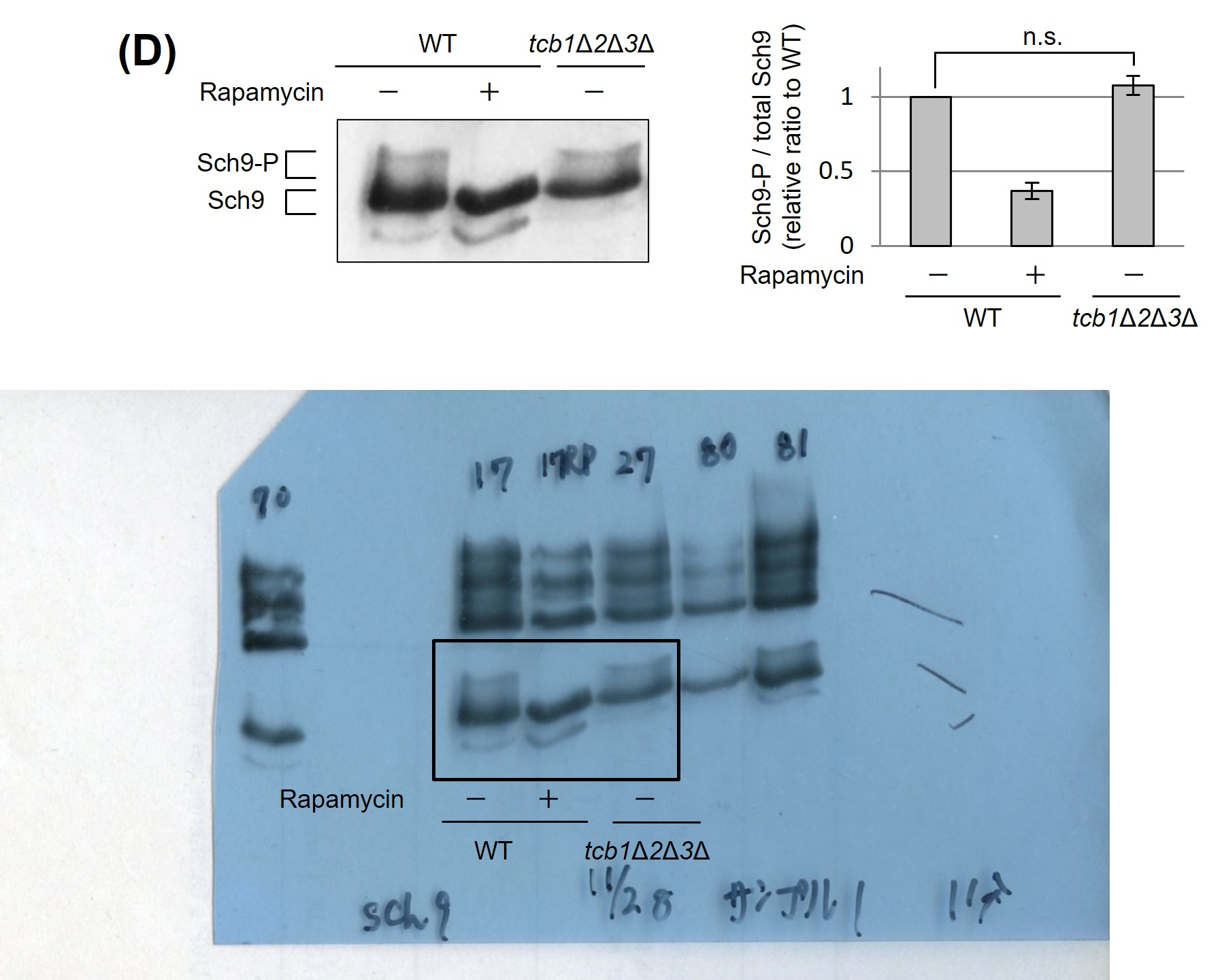

Supplement: Figure 1—source data 6. [file elife-89938-fig1-data6.zip › Figure 1-source data 6.jpg]

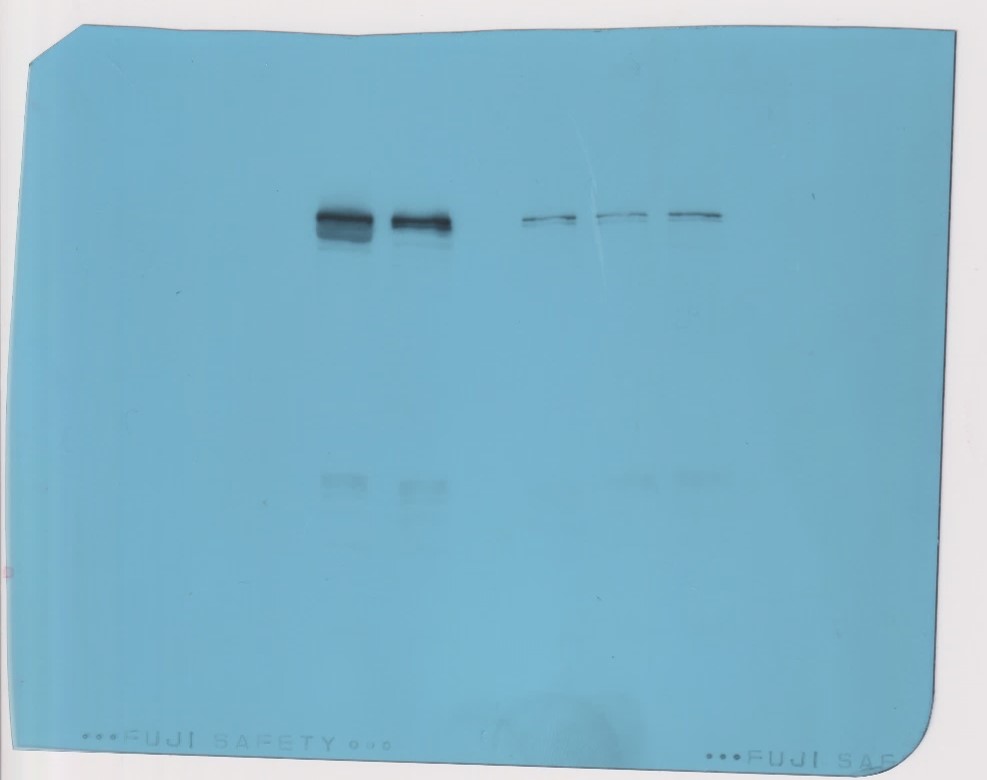

Supplement: Figure 2—figure supplement 1—source data 1. [file elife-89938-fig2-figsupp1-data1.zip › Figure 2-figure supplement 1-source data 1.jpg]

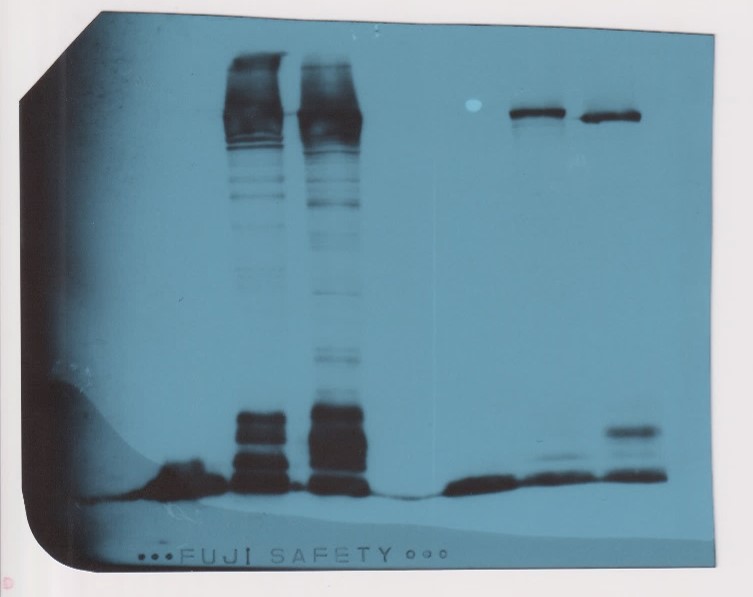

Supplement: Figure 2—figure supplement 1—source data 2. [file elife-89938-fig2-figsupp1-data2.zip › Figure 2-figure supplement 1-source data 2.jpg]

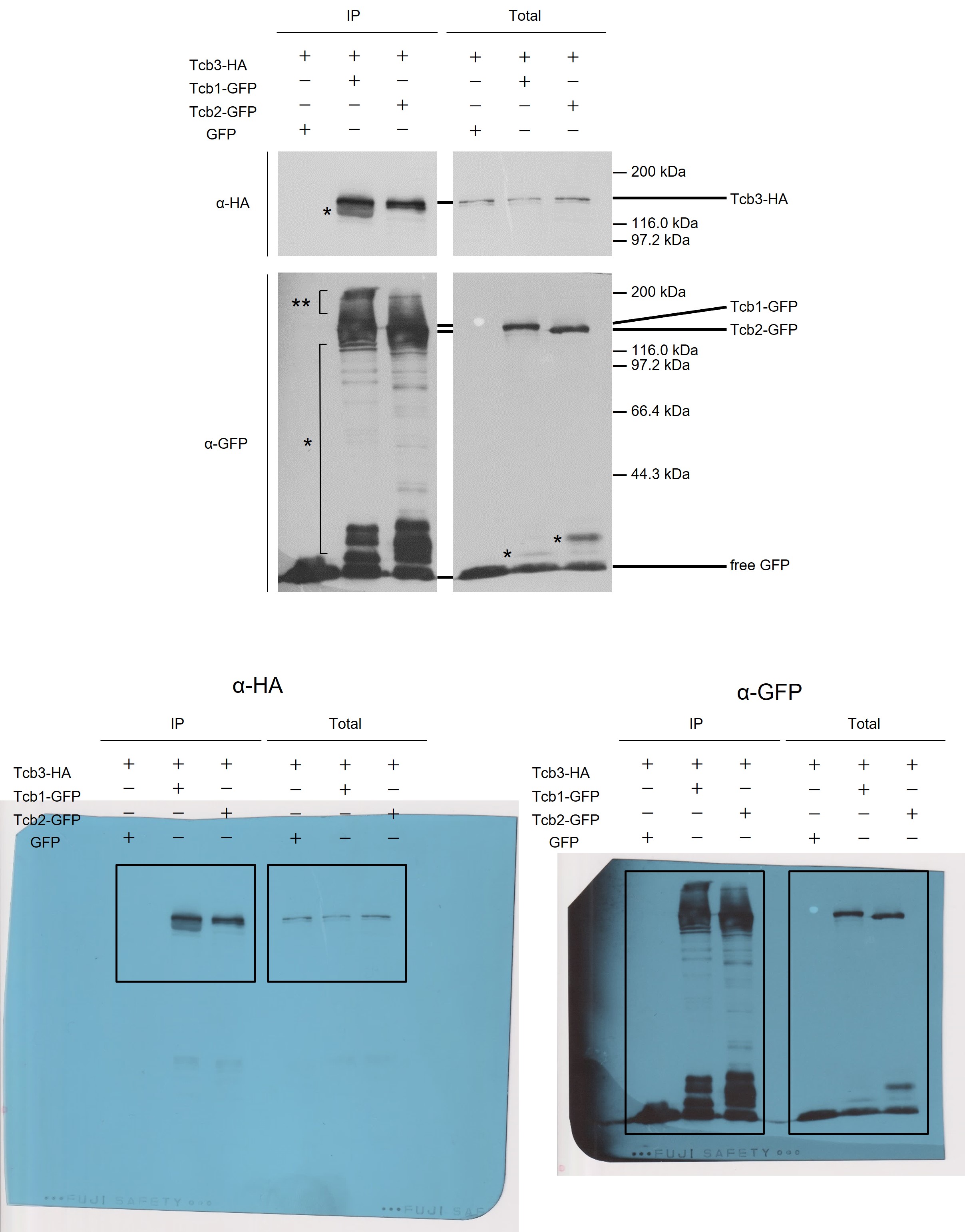

Supplement: Figure 2—figure supplement 1—source data 3. [file elife-89938-fig2-figsupp1-data3.zip › Figure 2-figure supplement 1-source data 3.jpg]

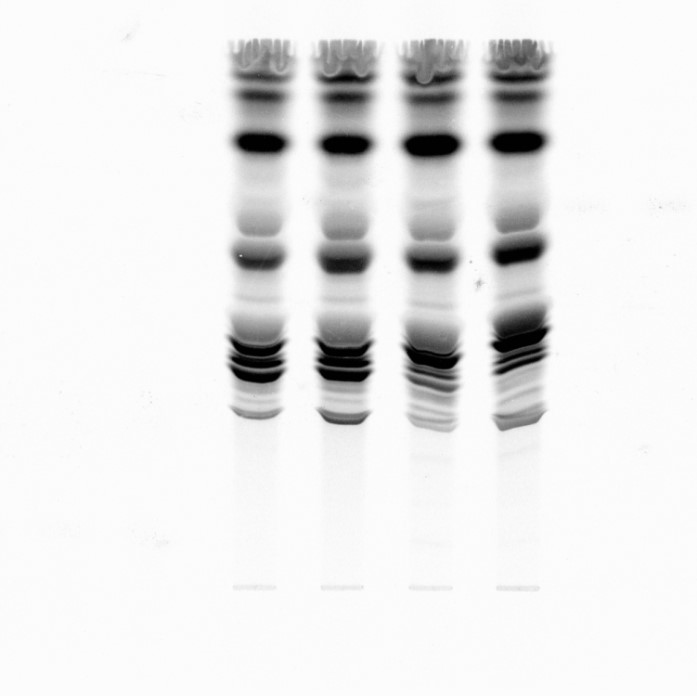

Supplement: Figure 3—source data 1. [file elife-89938-fig3-data1.zip › Figure 3-source data 1.jpg]

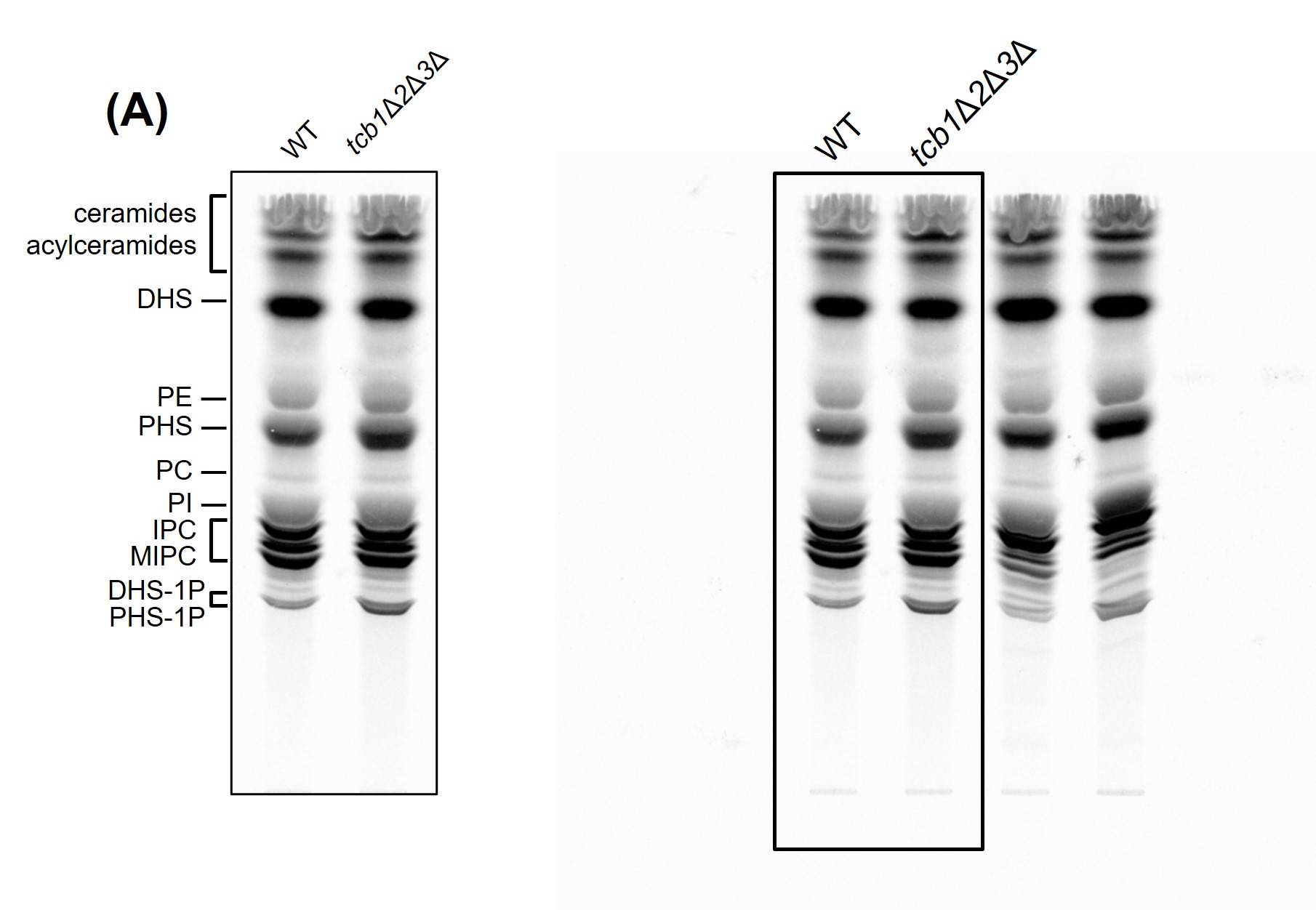

Supplement: Figure 3—source data 2. [file elife-89938-fig3-data2.zip › Figure 3-source data 2.jpg]

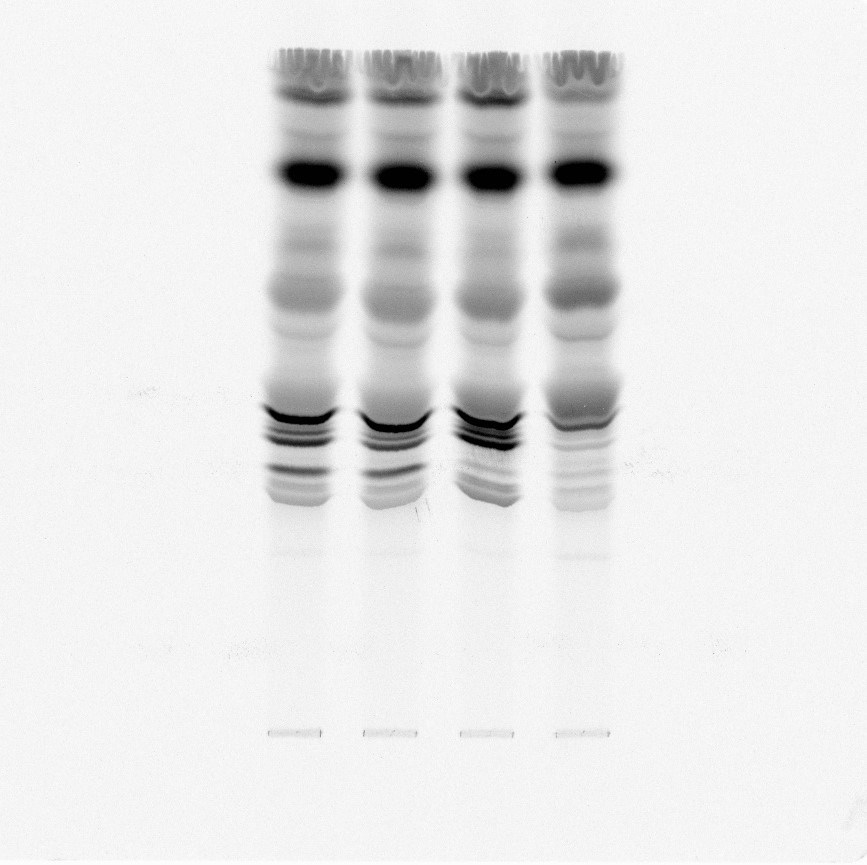

Supplement: Figure 5—source data 2. [file elife-89938-fig5-data2.zip › Figure 5-source data 2.jpg]

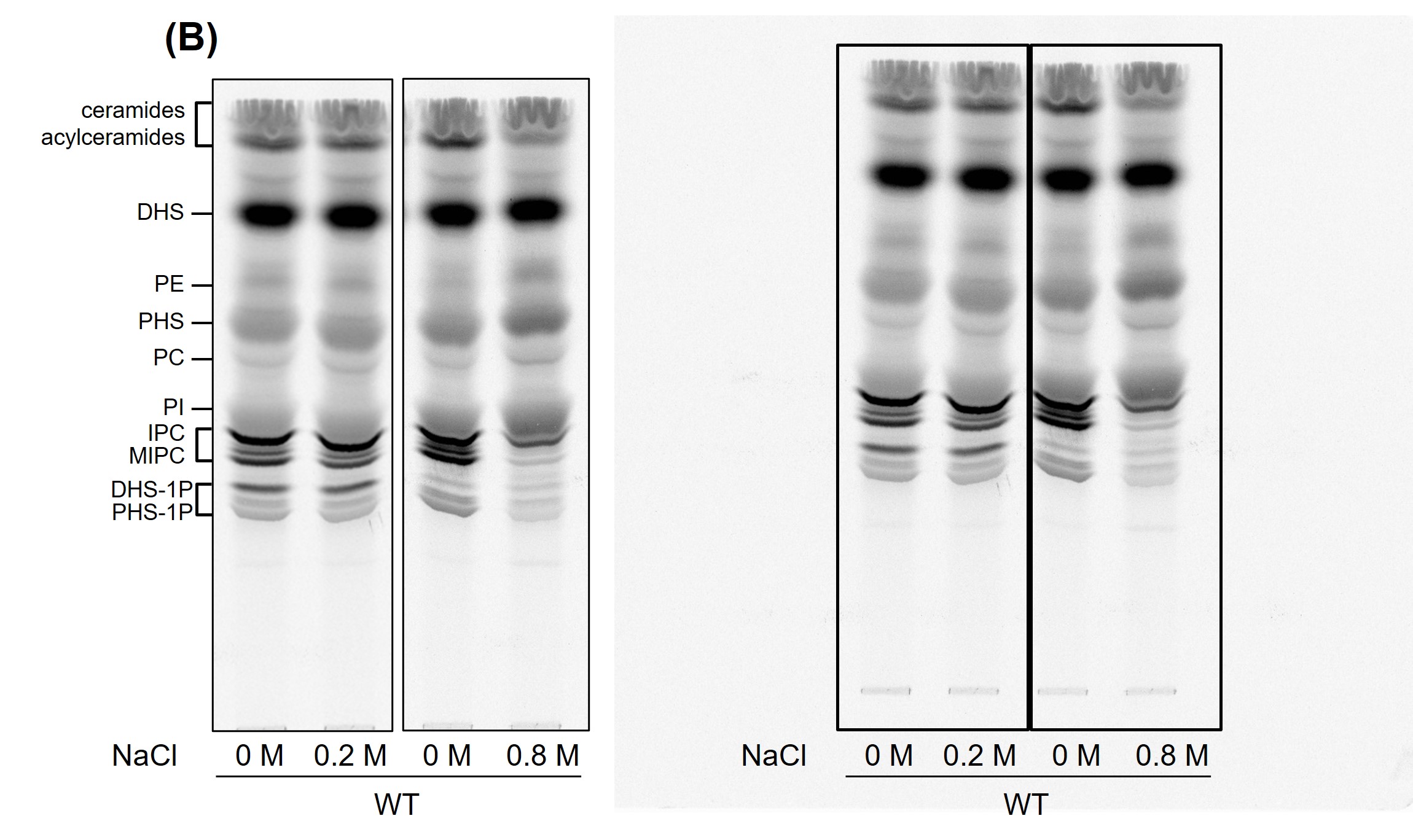

Supplement: Figure 5—source data 3. [file elife-89938-fig5-data3.zip › Figure 5-source data 3.jpg]
